# These Are Not the K-mers You Are Looking For: Efficient Online K-mer Counting Using a Probabilistic Data Structure

**DOI:** 10.1371/journal.pone.0101271

**Published:** 2014-07-25

**Authors:** Qingpeng Zhang, Jason Pell, Rosangela Canino-Koning, Adina Chuang Howe, C. Titus Brown

**Affiliations:** 1 Department of Computer Science and Engineering, Michigan State University, East Lansing, Michigan, United States of America; 2 Department of Microbiology and Molecular Genetics, Michigan State University, East Lansing, Michigan, United States of America; 3 Department of Plant, Soil, and Microbial Sciences, Michigan State University, East Lansing, Michigan, United States of America; Wayne State University, United States of America

## Abstract

K-mer abundance analysis is widely used for many purposes in nucleotide sequence analysis, including data preprocessing for de novo assembly, repeat detection, and sequencing coverage estimation. We present the khmer software package for fast and memory efficient *online* counting of k-mers in sequencing data sets. Unlike previous methods based on data structures such as hash tables, suffix arrays, and trie structures, khmer relies entirely on a simple probabilistic data structure, a Count-Min Sketch. The Count-Min Sketch permits online updating and retrieval of k-mer counts in memory which is necessary to support online k-mer analysis algorithms. On sparse data sets this data structure is considerably more memory efficient than any exact data structure. In exchange, the use of a Count-Min Sketch introduces a systematic overcount for k-mers; moreover, only the counts, and not the k-mers, are stored. Here we analyze the speed, the memory usage, and the miscount rate of khmer for generating k-mer frequency distributions and retrieving k-mer counts for individual k-mers. We also compare the performance of khmer to several other k-mer counting packages, including Tallymer, Jellyfish, BFCounter, DSK, KMC, Turtle and KAnalyze. Finally, we examine the effectiveness of profiling sequencing error, k-mer abundance trimming, and digital normalization of reads in the context of high khmer false positive rates. khmer is implemented in C++ wrapped in a Python interface, offers a tested and robust API, and is freely available under the BSD license at github.com/ged-lab/khmer.

## Introduction

The goal of k-mer counting is to determine the number of occurrences for each fixed-length word of length k in a DNA data set [1]. Efficient k-mer counting plays an important role in many bioinformatics approaches, including data preprocessing for de novo assembly, repeat detection, and sequencing coverage estimation [2].

Short-read shotgun sequencing data is both relatively sparse in k-mers and contains many erroneous k-mers. For typical values of k such as 32 these data sets are sparse, as only a small fraction of the total possible number of k-mers (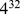) are actually present in any genome or read data sets derived from the genome. The high error rate (e.g. Illumina has a 0.1–1% per-base error rate [3]) generates many unique k-mers. As the total number of generated reads increases, the total number of errors grows with it linearly. This leads to data sets where the erroneous k-mers vastly outnumber the true k-mers [4]. Tracking and counting the resulting large number of k-mers, most of which are erroneous, has become an unavoidable and challenging task in sequence analysis [5].

A variety of k-mer counting approaches, and standalone software packages implementing them, have emerged in recent years; this includes Tallymer, Jellyfish, BFCounter, DSK, KMC, Turtle and KAnalyze [1], [2], [6]–[10].

These approaches and implementations each offer different algorithmic trade-offs and enable a non-overlapping set of functionality. Tallymer uses a suffix tree to store k-mer counts in memory and on disk [2]. Jellyfish stores k-mer counts in in-memory hash tables, and makes use of disk storage to scale to larger data sets [1]. BFCounter uses a Bloom filter as a pre-filter to avoid counting unique k-mers, and is the first published probabilistic approach to k-mer counting [6]. DSK adopts an approach to k-mer counting that enables time- and memory-efficient k-mer counting with an explicit trade-off between disk and memory usage [7]. KMC and KAnalyze rely primarily on fast and inexpensive disk access to count k-mers in low memory [8], [10]. Turtle provides several different containers that offer different false positive and false negative tradeoffs when counting k-mers [9].

Our motivation for exploring efficient k-mer counting comes from our work with metagenomic data, where we routinely encounter data sets that contain 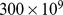 bases of DNA and over 50 billion distinct k-mers [11]. To efficiently filter, partition, and assemble these data, we need to store counts for each of these k-mers in main memory, and query and update them in realtime — a set of functionality not readily offered by current packages. Moreover, we wish to enable the use of cloud and desktop computers, which may have poor I/O performance or limited memory. These needs have dictated our exploration of efficient in-memory k-mer counting techniques.

Below, we describe an implementation of a simple probabilistic data structure for k-mer counting. This data structure is based on a Count-Min Sketch [12], a generalized probabilistic data structure for storing the frequency distributions of distinct elements. Our implementation extends an earlier implementation of a Bloom filter [13], which has been previously used in bioinformatics applications, such as sequence matching [14], k-mer counting [6], and de Bruijn graph storage and traversal [15], [16]. Many other variations of Bloom filters have been proposed [17], including counting Bloom filters [18], multistage filters [19], and spectral Bloom filters [20], which are related to the Count-Min Sketch and our khmer implementation.

Probabilistic approaches can be particularly memory efficient for certain problems, with memory usage significantly lower than any exact data structure [15]. However, their use introduces set membership or counting false positives, which have effects that must be analyzed in the context of specific problems. Moreover, unlike existing techniques, the Count-Min Sketch stores only counts; k-mers must be retrieved from the original data set. In exchange, the low memory footprint enabled by this probabilistic approach enables online updating and retrieval of k-mer counts entirely in memory, which in turn supports streaming applications such as digital normalization [21].

We use the Amazon cloud to compare time, memory, and disk usage of our k-mer counting implementation with that of other k-mer counting software packages, for two problems. First, we generate a k-mer abundance distribution for large data sets; and second, we query many individual k-mer counts at random from a previously constructed k-mer count database. We show that khmer is competitive in speed, memory, and disk usage for these problems. We also analyze the effects of counting error on calculations of the k-mer count in sequencing data sets, and in particular on metagenomic data sets. Finally, we discuss khmer's miscount performance in the context of two specific applications: low-abundance k-mer trimming of reads, and digital normalization.

The khmer software [22] is implemented in C++ in a Python wrapper, enabling flexible use and reuse by users with a wide range of computational expertise. The software package is freely available for academic and commercial use and redistribution under the BSD license at github.com/ged-lab/khmer/. khmer comes with substantial documentation and many tutorials, and contains extensive unit tests. Moreover, we have built several applications on top of khmer, including memory-efficient de Bruijn graph partitioning [15] and lossy compression of short-read data sets for assembly [21].

## Results

### Implementing a Count-Min Sketch for k-mers

The two basic operations supported by khmer are increment_count(kmer) and c =  get_count(kmer). Both operate on the data structure in memory, such that neither incrementing a count nor retrieving a count involves disk access.

The implementation details are similar to those of the Bloom filter in [15], but with the use of 8 bit counters instead of 1 bit counters. Briefly, Z hash tables are allocated, each with a different size of approximately H bytes (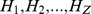); the sum of these hash table sizes must fit within available main memory. To increment the count for a particular k-mer, a single hash is computed for the k-mer, and the modulus of that hash with each hash table's size H gives the location for each hash table; the associated count in each hash table is then incremented by 1. We use different sizes for each hash table so as to vary the hash function. Even if two k-mers have the same modulus in one hash table (a collision), they are unlikely to collide in the other hash tables. To retrieve the count for a k-mer, the same hash is computed and the minimum count across all hash tables is computed. While different in implementation detail from the standard Bloom filter, which uses a single hash table with many hash functions, the performance details are identical [15]. One particularly important feature of the Count-Min Sketch is that the counting error is *one-sided* [12]. Because counts are only incremented, collisions result in inflated miscounts; if there is no collision for a particular k-mer, the count is correct.

An additional benefit of the Count-Min Sketch is that it is extremely easy to implement correctly, needing only about 3 dozen lines of C++ code for a simple threadsafe implementation. (We have described how khmer scales with multiple threads in [23].)

To determine the expected false positive rate — the average frequency with which a given k-mer count will be incorrect when retrieved — we can look at the hash table load. Suppose N distinct k-mers have been counted using Z hash tables, each with size H. The probability that no collisions happened in a specific entry in one hash table is 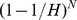, or approximately 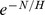. The individual collision rate in one hash table is then 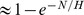. The total collision rate, which is the probability that a collision occurred in each entry where a k-mer maps across all Z hash tables, is 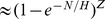, which is also the expected false positive rate.

While the false positive rate can easily be calculated from the hash table load, the average *miscount* — the degree to which the measured count differs from the true count — depends on the k-mer frequency distribution, which must be determined empirically. We analyze the effects of this below.

### Choosing number and size of hash tables used for k-mer counting

The false positive rate depends on the number of distinct k-mers 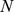, the number of hash tables 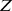, and the size of the hash tables 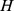: 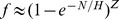, with an associated memory usage of 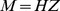. We face two common scenarios: one in which we have a fixed number of k-mers 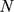 and fixed memory 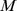 and we want to calculate the optimal number of hash tables 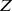; and one in which we have a desired maximum false positive rate 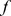 and a fixed number of k-mers 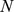, and we want to calculate the minimum memory usage required to achieve 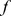.

For fixed memory 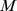 and number of distinct k-mers 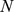, the optimal number of hash tables can be found by minimizing 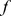; taking the derivative, 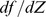, with 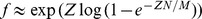 and solving for 0, we find that 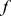 is minimized when 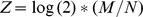 (see [24] for details).

Given a desired false positive rate 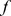 and a fixed number of k-mers 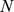, the optimal memory usage can be calculated as follows. First, the optimal number of hash tables is determined by the expected false positive rate alone: 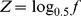. Using this 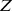, the minimum average hash table size 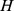 necessary to achieve 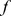 can be calculated as 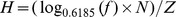 (see [24] for details).

A remaining problem is that the number of distinct k-mers 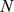 is typically not known. However, memory- and time-efficient algorithms for calculating 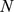 do exist and we plan to implement this in khmer in the future [25].

### khmer efficiently calculates k-mer abundance histograms

**Figure 1:**
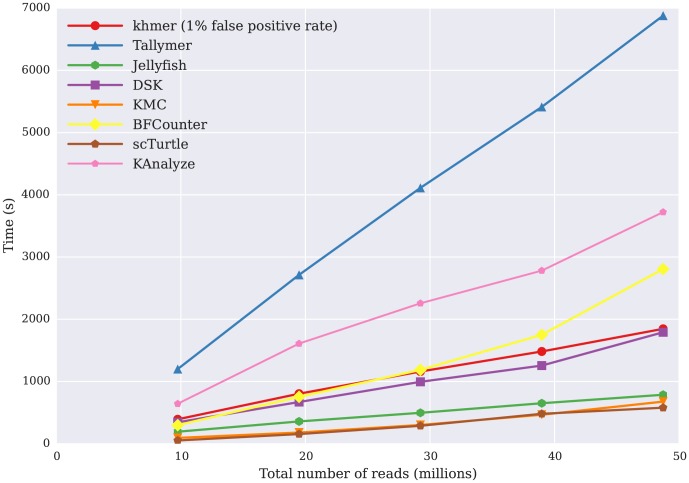
Comparison of the time it takes for k-mer counting tools to calculate k-mer abundance histograms, with time (y axis, in seconds) against data set size (in number of reads, x axis). All programs executed in time approximately linear with the number of input reads.

**Figure 2:**
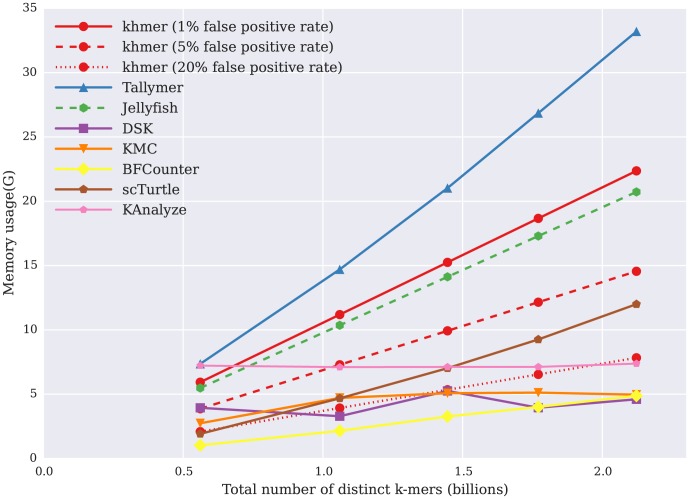
Memory usage of k-mer counting tools when calculating k-mer abundance histograms, with maximum resident program size (y axis, in GB) plotted against the total number of distinct k-mers in the data set (x axis, billions of k-mers).

We measured time and memory required to calculate k-mer abundance histograms in five soil metagenomic read data sets using khmer, Tallymer, Jellyfish, DSK, KMC, Turtle, and KAnalyze (Table 1; Figures 1 and 2). We chose to benchmark abundance histograms because this functionality is common to all the software packages, and is a common analysis approach for determining assembly parameters [26]. We applied each package to increasingly large subsets of a 50 m read soil metagenome data set [11]. For the BFCounter, KMC, Turtle and KAnalyze packages, which do not generate k-mer abundance distribution directly, we output the frequency of each k-mer to a file but do no further analysis.

**Table 1:** Benchmark soil metagenome data sets for k-mer counting performance, taken from [11].

Data set	size of file (GB)	number of reads	number of distinct k-mers	total number of k-mers
subset 1	1.90	9,744,399	561,178,082	630,207,985
subset 2	2.17	19,488,798	1,060,354,144	1,259,079,821
subset 3	3.14	29,233,197	1,445,923,389	1,771,614,378
subset 4	4.05	38,977,596	1,770,589,216	2,227,756,662
entire data set	5.00	48,721,995	2,121,474,237	2,743,130,683

Figure 1 shows that the time usage of the khmer approach is comparable to DSK and BFCounter, and, as expected, increases linearly with data set size. Tallymer is the slowest of the four tools in this testing, while KMC, Turtle, and Jellyfish are the fastest.

From Figure 2, we see that the memory usage of Jellyfish, Tallymer, BFCounter, and Turtle increases linearly with data set size. Tallymer uses more memory than Jellyfish generally, while BFCounter and Turtle have considerably lower memory usage. DSK, KMC, and KAnalyze use constant memory across the data sets, but at the cost of more limited functionality (discussed below).

The memory usage of khmer also increases linearly with data set size as long as we hold the false positive rate constant. However, the memory usage of khmer varies substantially with the desired false positive rate: we can decrease the memory usage by increasing the false positive rate as shown in Figure 2. We also see that with a low false positive of 1%, the memory usage is competitive with Tallymer and Jellyfish; with a higher 5% false positive rate, the memory usage is lower than all but the disk-based DSK; with an false positive rate as high as 20%, the memory usage is further lower, close to DSK, KAnalyze, and KMC.

**Figure 3:**
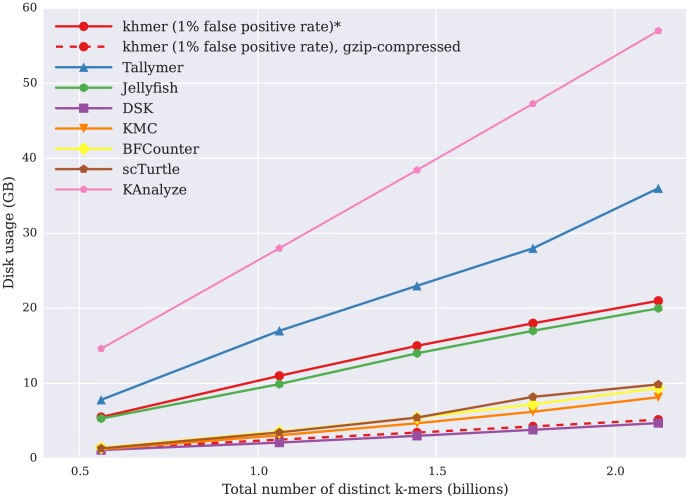
Disk storage usage of different k-mer counting tools to calculate k-mer abundance histograms in GB (y axis), plotted against the number of distinct k-mers in the data set (x axis). 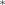
Note that khmer does not use the disk during counting or retrieval, although its hash tables can be saved for reuse.

We also measured disk usage during counting. Figure 3 shows that the disk usage also increases linearly with the number of k-mers in the data set. For a high-diversity metagenomic data set of 5 GB, the disk usage of both Jellyfish and Tallymer is around 30 GB. khmer counts k-mers entirely in working memory and does not rely on any on-disk storage to store or retrieve k-mer counts, although for practicality the hash tables can be saved for later reuse; the uncompressed disk usage for khmer in Figure 3 is the same as its memory. At the expense of more time, khmer supports saving and loading gzip-compressed hash tables, which are competitive in size to DSK's on-disk database (Figure 3, dashed line).

### khmer accesses k-mer counts efficiently

**Figure 4:**
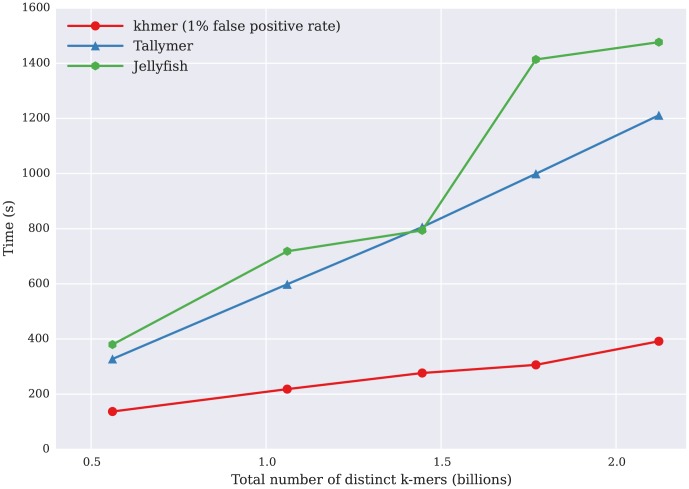
Time for several k-mer counting tools to retrieve the counts of 9.7 m randomly chosen k-mers (y axis), plotted against the number of distinct k-mers in the data set being queried (x axis). BFCounter, DSK, Turtle, KAnalyze, and KMC do not support this functionality.

We measured the time it took to access 9.7 m 22-mers across five different data sets after the initial databases had been built (Figure 4). Note that Tallymer, Jellyfish, and khmer all support random access to k-mer counts, while BFCounter, DSK, KMC, Turtle and KAnalyze do not. Here, khmer performed well, dramatically outperforming Jellyfish and Tallymer. In all three cases, system time dominated the overall time required to retrieve k-mers, suggesting that the primary reason for the increase in retrieval time was due to the increased size of the database on the disk (data not shown). In particular, khmer is independent of the size of the database in retrieval time once the hash tables are loaded into memory.

### The measured counting error is low on short-read data

Due to the use of Count-Min Sketch and its lack of collision tracking, khmer will report some incorrect counts for k-mers; these counts are always higher than the true counts, up to the bound of 255 (a limit imposed by our use of 8-bit counters). The frequency with which incorrect counts are reported can be estimated from the hash table load. However, the expected *miscount* — the difference between the true k-mer frequency and the reported k-mer frequency — cannot be calculated without knowing the distribution of k-mer abundances; in general, the average miscount will be small if the data is left-skewed. As noted by Melsted and Pritchard, a large number of k-mers in short-read data are low-abundance, leading to precisely the skew that would yield low miscounts [6]. Here we use both real and simulated data sets to evaluate the counting performance in practice.

**Figure 5:**
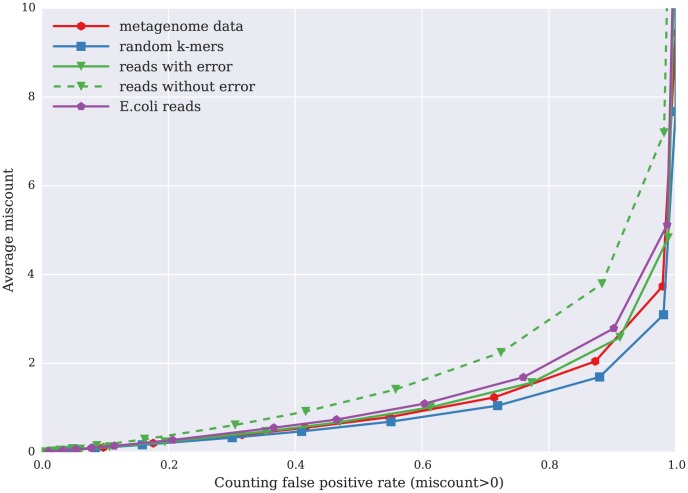
Relation between average miscount — amount by which the count for k-mers is incorrect — on the y axis, plotted against false positive rate (x axis), for five data sets. The five data sets were chosen to have the same total number of distinct k-mers: one metagenome data set; a set of randomly generated k-mers; a set of reads, chosen with 3x coverage and 1% error, from a randomly generated genome; a simulated set of error-free reads (3x) chosen from a randomly generated genome and a set of *E. coli* reads.

Figure 5 shows the relationship between average miscount and counting false positive rate for five different test data sets with similar numbers of distinct k-mers: one metagenome data set; a simulated set of random k-mers; a simulated set of reads, chosen with 3x coverage and 1% error; a simulated set of reads (3x) with no error; and a set of *E. coli* reads (Table 2). Even when the counting false positive rate is as high as 0.9 — where 90% of k-mers have an incorrect count — the average miscount is still below 4.

**Table 2:** Data sets used for analyzing miscounts.

Data set	Size of data set file	Number of total k-mers	Number of distinct k-mers
Real metagenomics reads	7.01 M	2,917,200	1,944,996
Totally random reads with randomly generated k-mers	3.53 M	2,250,006	1,973,059
Simulated reads from simulated genome with error	5.92 M	3,757,479	2,133,592
Simulated reads from simulated genome without error	9.07 M	5,714,973	1,989,644
Real *E. coli* reads	4.85 M	4,004,911	2,079,302

**Figure 6:**
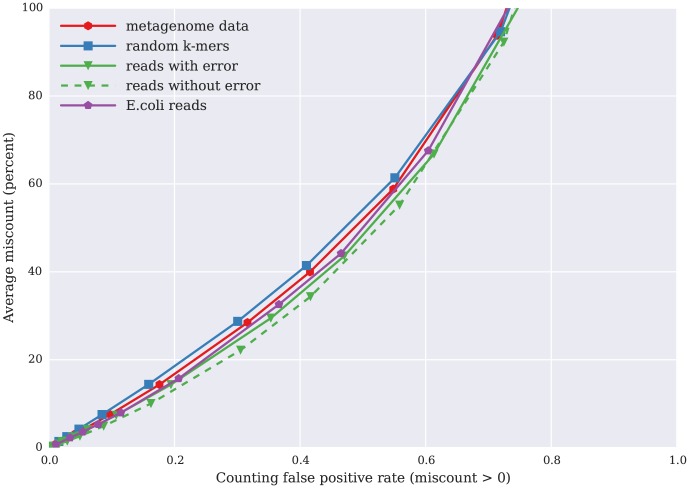
Relation between percent miscount — amount by which the count for k-mers is incorrect relative to its true count — on the y axis, plotted against false positive rate (x axis), for five data sets. The five data sets are the same as in Figure 5.

We separately analyzed the average *percentage* miscount between true and false k-mers; e.g. an miscount of 4 for a k-mer whose true count is 1 would be 400%. Figure 6 shows the relationship between average miscount and counting false positive rate for the same five data sets as in Figure 5. For a false positive rate of 0.1 (10% of k-mer counts are incorrect), the average percentage miscount is less than 10% for all five data sets; this will of course generally be true, because the average miscount is bounded by the product of the false positive rate with k-mer abundance.

We see here that for a fixed false positive rate, the simulated reads without error have the highest average miscount, and the randomly generated k-mers have the lowest average miscount. This is because these two abundance distributions have the least and most left-skew, respectively: the simulated reads without error have no abundance-1 k-mers, while the randomly generated k-mers are entirely low abundance.

### Sequencing error profiles can be measured with k-mer abundance profiles

One specific use for khmer is detecting random sequencing errors by looking at the k-mer abundance distribution within reads [27]. This approach, known also as “k-mer spectral analysis”, was first proposed in by [28] and further developed in [29]. The essential idea is that low-abundance k-mers contained in a high-coverage data set typically represent random sequencing errors.

A variety of read trimming and error correcting tools use k-mer counting to reduce the error content of the read data set, independent of quality scores or reference genomes [30]. This is an application where the counting error of the Count-Min Sketch approach used by khmer may be particularly tolerable: it will never falsely call a high-abundance k-mer as low-abundance because khmer never underestimates counts.

**Figure 7:**
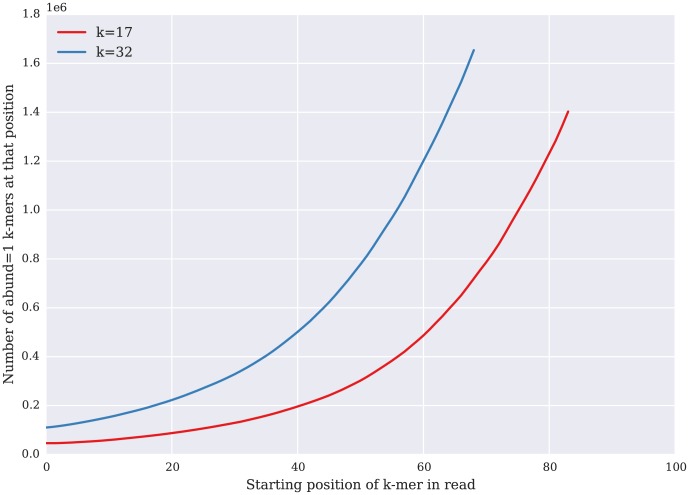
Number of unique k-mers (y axis) by starting position within read (x axis) in an untrimmed *E. coli* 100-bp Illumina shotgun data set, for k = 17 and k = 32. The increasing numbers of unique k-mers are a sign of the increasing sequencing error towards the 3′ end of reads. Note that there are only 69 starting positions for 32-mers in a 100 base read.

In Figure 7, we use khmer to examine the sequencing error pattern of a 5m-read subset of an Illumina reads data set from single-colony sequencing of *E. coli* [31]. The high rate of occurrence of unique k-mers close to the 3′ end of reads is due to the increased sequencing error rate at the 3′ end of reads.

### khmer can be applied iteratively to read trimming

We next evaluated the effect of false-positive induced miscounts on read trimming, in which reads are truncated at the first low-abundance k-mer. Because the Count-Min Sketch never undercounts k-mers, reads will never be erroneously trimmed at truly high-abundance k-mers; however, reads may not be trimmed correctly when miscounts inflate the count of low-abundance k-mers. In cases where many errors remain, read trimming can potentially be applied multiple times, with each round reducing the total number of k-mers and hence resulting in lower false positive rates for the same memory usage.

We performed six iterations of unique k-mer trimming on 5 million Illumina reads from sequencing of *E. coli*, with memory usage less than 30 MB. For each iteration we measured empirical false positive rate compared with number of bases trimmed as well as the total number of k-mers (Table 3). In the first round, the estimated false positive rate was 80.0%, and 13.5% of the total bases were removed by trimming reads at low-abundance k-mers; the second iteration had a false positive rate of 37.7%, and removed only 1.5% additional data; and by the fourth iteration the false positive rate was down to 23.2% with 0.0% of the data removed.

**Table 3:** Iterative low-memory k-mer trimming.

	FP rate	bases trimmed	distinct k-mers	unique k-mers	unique k-mers at 3′ end
untrimmed	-	-	41.6 m	34.1 m	30.4%
khmer iteration 1	80.0%	13.5%	13.3 m	6.5 m	29.8%
khmer iteration 2	40.2%	1.7%	7.6 m	909.9k	12.3%
khmer iteration 3	25.4%	0.3%	6.8 m	168.1k	3.1%
khmer iteration 4	23.2%	0.1%	6.7 m	35.8k	0.7%
khmer iteration 5	22.8%	0.0%	6.6 m	7.9k	0.2%
khmer iteration 6	22.7%	0.0%	6.6 m	1.9k	0.0%
filter by FASTX	-	9.1%	26.6 m	20.3 m	26.3%
filter by seqtk(default)	-	8.9%	17.7 m	12.1 m	12.3%
filter by seqtk(-q 0.01)	-	15.4%	9.9 m	5.1 m	5.2%
filter by seqtk(-b 3 -e 5)	-	8.0%	34.5 m	27.7 m	25.3%

**The results of trimming reads at unique (erroneous) k-mers from a 5 m read** ***E. coli*** **data set (1.4 GB) in under 30 MB of RAM. After each iteration, we measured the total number of distinct k-mers in the data set, the total number of unique (and likely erroneous) k-mers remaining, and the number of unique k-mers present at the 3' end of reads.**

The elimination of so many unique k-mers (column 5) in the first pass was unexpected: the high false positive rate should have resulted in fewer k-mers being identified as unique, were the erroneous k-mers independent of each other. Upon examination, we realized that in Illumina data erroneous k-mers typically come from substitution errors that yield runs of up to k erroneous k-mers in a row [30]. When trimming reads with high false positive rates, these runs are typically trimmed after the first few unique k-mers, leaving unique k-mers at the 3′ end. Because of this we hypothesized that high-FP rate trimming would result in the retention of many unique k-mers at the 3′ end of the read, and this was confirmed upon measurement (Table 3, column 6, pass 1 vs pass 2).

In comparison to quality-based trimming software such as seqtk and FASTX, trimming at unique k-mers performed very well: in this data set, all unique k-mers represent errors, and even with an initial false positive rate of 80%, khmer outperformed all but the most stringent seqtk run (Table 3). With a lower false positive rate or multiple passes, khmer eliminates more erroneous k-mers than seqtk or FASTX. The tradeoff here is in memory usage: for larger data sets, seqtk and FASTX will consume the same amount of memory as on smaller data sets, while khmer's memory usage will need to grow with the data set size.

### Using khmer for digital normalization, a streaming algorithm

Digital normalization is a lossy compression algorithm that discards short reads based on saturating coverage of a de Bruijn graph [21]. While several non-streaming implementations exist, including Trinity's *in silico* normalization [32], [33], digital normalization can be efficiently implemented as a *streaming* algorithm. In the streaming implementation, if a read is not kept, it is not loaded into the Count-Min Sketch structure, and the false positive rate does not increase. For high coverage data sets, the digital normalization algorithm is sublinear in memory because it does not collect the majority of k-mers in those data sets [21]. This has the advantage of enabling low-memory preprocessing of both high-coverage genomic data sets, as well as mRNAseq or metagenomic data sets with high-coverage components [11], [21].

While digital normalization is already implemented inside khmer, previous work did not explore the lower bound on memory usage for effective digital normalization. In particular, the effects of high false positive rates have not been examined in any prior work.

We applied digital normalization to the *E. coli* data set used above, and chose seven different Count-Min Sketch sizes to yield seven different false positive rates 4. The data set was normalized to a k-mer coverage of 20 and the resulting data were evaluated for retention of true and erroneous k-mers, as in [21] (Table 4). The results show that digital normalization retains the same set of underlying “true” k-mers until the highest false positive rate of 100% (Table 4, column 5), while discarding only about 2% additional reads (Table 4, column 6).

**Table 4:** Low-memory digital normalization.

memory	FP rate	retained reads	retained reads %	true k-mers missing	total k-mers
before diginorm	-	5,000,000	100.0%	170	41.6 m
2400 MB	0.0%	1,656,518	33.0%	172	28.1 m
240 MB	2.8%	1,655,988	33.0%	172	28.1 m
120 MB	18.0%	1,652,273	33.0%	172	28.1 m
60 MB	59.1%	1,633,182	32.0%	172	27.9 m
40 MB	83.2%	1,602,437	32.0%	172	27.6 m
20 MB	98.8%	1,460,936	29.0%	172	25.7 m
10 MB	100.0%	1,076,958	21.0%	185	20.9 m

**The results of digitally normalizing a 5 m read** ***E. coli*** **data set (1.4 GB) to C = 20 with k = 20 under several memory usage/false positive rates. The false positive rate (column 1) is empirically determined. We measured reads remaining, number of “true” k-mers missing from the data at each step, and the number of total k-mers remaining. Note: at high false positive rates, reads are erroneously removed due to inflation of k-mer counts.**

To evaluate the effect of digital normalization with high false positive rates on actual genome assembly, we next performed normalization to a coverage of 20 with the same range of false positive rates as above. We then assembled this data with Velvet [34] and compared the resulting assemblies to the known *E. coli* MG1655 genome using QUAST (Table 5). To our surprise, we found that even after executing digital normalization with a false positive rate of 83.2%, a nearly complete assembly was generated. No progressive increase in misassemblies (measured against the real genome with QUAST) was seen across the different false positive rates (data not shown). This suggests that below 83.2% FP rate, the false positive rate of digital normalization has little to no effect on assembly quality with Velvet. (Note that the Velvet assembler itself used considerably more memory than digital normalization.)

**Table 5:** *E. coli* genome assembly after low-memory digital normalization.

memory	FP rate	N contigs	total length(bases)	% of true genome covered
before diginorm	-	106	4,546,051	97.84%
2400 MB	0.0%	617	4,549,235	98.05%
240 MB	2.8%	87	4,549,253	98.04%
120 MB	18.0%	86	4,549,335	98.04%
60 MB	59.1%	90	4,548,619	98.03%
40 MB	83.2%	89	4,550,599	98.11%
20 MB	98.8%	85	4,550,014	98.04%
10 MB	100.0%	97	4,545,871	97.97%

**A comparison of assembling reads digitally normalized with low memory/high false positive rates. The reads were digitally normalized to C = 20 (see [21] for more information) and were assembled using Velvet. We measured total length of assembly, as well as percent of true MG1655 genome covered by the assembly using QUAST.**

While these results are specific to Velvet and the coverage parameters used in digital normalization, they do suggest that no significant information loss occurs due to false positive rates below 80%. Further evaluation of assembly quality in response to different normalization parameters and assemblers is beyond the scope of of this paper.

## Discussion

### khmer enables fast, memory-efficient online counting

khmer enables memory- and time-efficient online counting (Figures 1, 2, and 4). This is particularly important for the streaming approaches to data analysis needed as data set sizes increase. Because query and updating of k-mer counts can be done directly as data is being loaded, with no need for disk access or an indexing step, khmer can also perform well in situations with poor disk I/O performance. (Note that BFCounter also supports online k-mer counting [6].)

### khmer is a generally useful k-mer counting approach

In addition to online counting, khmer offers a general range of useful performance tradeoffs for disk I/O, time and memory. From the performance comparison between khmer and other k-mer counting packages in calculating k-mer abundance distributions, khmer is comparable with existing packages. In time, khmer performs competitively with DSK and BFCounter (Figure 1); khmer also provides a way to systematically trade memory for miscounts across a wide range of parameters (Figure 2). khmer's uncompressed disk storage is competitive with Jellyfish, and, in situations where disk space is at a premium, khmer can take advantage of gzip compression to provide storage similar to that of DSK (Figure 3, purple line with boxes).

KMC, DSK, and KAnalyze perform especially well in memory usage for calculating the abundance distribution of k-mers. However, in exchange for this efficiency, retrieving specific k-mer counts at random is likely to be quite slow, as DSK is optimized for iterating across partition sets of k-mers rather than randomly accessing k-mer counts.

For retrieving the counts of individual k-mers, khmer is significantly faster than both Tallymer and Jellyfish. This is not surprising, since this was a primary motivation for the development of khmer.

### khmer memory usage is fixed and low

The memory usage of the basic Count-Min Sketch approach is fixed: khmer's memory usage does not increase as data is loaded. While this means that khmer will never crash due to memory limitations, and all operations can be performed in main memory without recourse to disk storage, the false positive rate may grow too high. Therefore the memory size must be chosen in light of the false positive rate and miscount acceptable for a given application. In practice, we recommend choosing the maximum available memory, because the false positive rate decreases with increasing memory and there are no negative effects to minimizing the false positive rate.

For any given data set, the size and number of hash tables will determine the accuracy of k-mer counting with khmer. Thus, the user can control the memory usage based on the desired level of accuracy (Figure 2). The time usage for the first step of k-mer counting, consuming the reads, depends on the total amount of data, since we must traverse every k-mer in every read. The second step, k-mer retrieval, is algorithmically constant for fixed k; however, for practicality, the hash tables are usually saved to and loaded from disk, meaning that k-mer retrieval time depends directly on the size of the database being queried.

The memory usage of khmer is particularly low for sparse data sets, especially since only main memory is used and no disk space is necessary beyond that required for the read data sets. This is no surprise: the information theoretic comparison in [15] shows that, for sparse sequencing data sets, Bloom filters require considerably less memory than any possible exact information storage for a wide range of false positive rates and data set sparseness.

In our implementation we use 1 byte to store the count of each k-mer in the data structure. Thus the maximum count for a k-mer will be 255. In cases where tracking bigger counts is required, khmer also provides an option to use an STL map data structure to store counts above 255, with the trade-off of significantly higher memory usage. In the future, we may extend khmer to counters of arbitrary bit sizes.

### False positive rates in k-mer counting are low and predictable

The Count-Min Sketch is a probabilistic data structure with a one-sided error that results in random overestimates of k-mer frequency, but does not generate underestimates.

In the Count-Min Sketch, the total memory usage is fixed; the memory usage, the hash functions, and the total number of distinct objects counted all influence the accuracy of the count. While the probability of an inaccurate count can easily be estimated based on the hash table load, the miscount size is dependent on details of the frequency distribution of k-mers [12].

More specifically, in the analysis of the Count-Min Sketch, the difference between the incorrect count and actual count is related to the total number of k-mers in a data set and the size of each hash table [12]. Further study has shown that the behavior of Count-Min Sketch depends on specific characteristics of the data set under consideration, especially left-skewness [35], [36]. These probabilistic properties suit short reads from next generation sequencing data sets: the miscounts are low because of the highly left-skewed abundance distribution of k-mers in these data sets.

Figures 5 and 6 demonstrate these properties well. We see more correct counting for error-prone reads from a genome than for error-free reads from a genome, with a normal distribution of k-mer abundance. Thus, this counting approach is especially suitable for high diversity data sets, such as metagenomic data, in which a larger proportion of k-mers are low abundance or unique due to sequencing errors.

### Real-world applications for khmer

For many applications, an approximate k-mer count is sufficient. For example, when eliminating reads with low abundance k-mers, we can tolerate a certain number of low-frequency k-mers remaining in the resulting data set falsely. If RAM-limited we can do the filtering iteratively so that at each step we are making more effective use of the available memory.

In practice, we have found that a false positive rate of between 1% and 10% offers acceptable miscount performance for a wide range of tasks, including error profiling, digital normalization and low-abundance read-trimming. Somewhat surprisingly, false positive rates of up to 80% can still be used for both read trimming and digital normalization in memory-limited circumstances, although multiple passes across the data may be needed.

For many applications, the fact that khmer does not break an imposed memory bound is extremely useful, since for many data sets — especially metagenomic data sets — high memory demands constrain analysis [11], [37]. Moreover, because the false positive rate is straightforward to measure, the user can be warned that the results should be invalidated when too little memory is used. When combined with the graceful degradation of performance for both error trimming and digital normalization, khmer readily enables analysis of extremely large and diverse data sets [38]. In an experiment to assemble the reads of a soil metagenomic sample collected from Iowa prairie, the number of reads to assemble drops from 3.3 million to 2.2 million and the size of the data set drops from 245GB to 145GB accordingly after digital normalization [11]. 240GB memory was used in the process. This also shows that khmer works well to analyze large, real-world metagenomic data sets.

### Conclusion

K-mer counting is widely used in bioinformatics, and as sequencing data set sizes increase, graceful degradation of data structures in the face of large amounts of data has become important. This is especially true when the theoretical and practical effects of the degradation can be predicted (see e.g. [6], [9], [15]). This is a key property of the Count-Min Sketch approach, and its implementation in khmer.

The khmer software implementation offers good performance, a robust and well-tested Python API, and a number of useful and well-documented scripts. While Jellyfish, DSK, KMC, and Turtle also offer good performance, khmer is competitive, and, because it provides a Python API for online counting, is flexible. In memory-limited situations with poor I/O performance, khmer is particularly useful, because it will not break an imposed memory bound and does not require disk access to store or retrieve k-mer counts. However, in exchange for this memory guarantee, counting becomes increasingly incorrect as less memory is used or as the data set size grows large; in many situations this may be an acceptable tradeoff.

### Future considerations

Applying khmer to extremely large data sets with many distinct k-mers requires a large amount of memory: approximately 446 GB of memory is required to achieve an false positive rate of 1% for 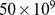 k-mers. It is possible to reduce the required memory by dividing k-mer space into multiple partitions and counting k-mers separately for each partition. Partitioning k-mer space into 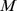 partitions results in a linear decrease in the number of k-mers under consideration, thus reducing the occupancy by a constant factor 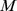 and correspondingly reducing the collision rate. Partitioning k-mer space is a generalization of the systematic prefix filtering approach, where one might first count all k-mers starting with AA, then AC, then AG, AT, CA, etc., which is equivalent to partitioning k-mer space into 16 equal-sized partitions. These partitions can be calculated independently, either across multiple machines or iteratively on a single machine, and the results stored for later comparison or analysis. This is similar to the approach taken by DSK [7], and could easily be implemented in khmer.

Further optimization of khmer on single machines, e.g. for multi-core architectures, is unlikely to achieve significantly greater speed. Past a certain point k-mer counting is fundamentally I/O bound [23].

Perhaps the most interesting future direction for probabilistic k-mer counting is that taken by Turtle [9], in which several data structures are provided, each with different tradeoffs, but with a common API. We hope to pursue this direction in the future by integrating such approaches into khmer.

## Methods

### Code and data set availability

The version of khmer used to generate the results below is available at http://github.com/ged-lab/khmer.git, tag ‘2013-khmer-counting’. Scripts specific to this paper are available in the paper repository at https://github.com/ged-lab/2013-khmer-counting. The IPython[39] notebook file and data analysis to generate the figures are also available in that github repository. Complete instructions to reproduce all of the results in this paper are available in the khmer-counting repository; see README.rst.

### Sequence Data

One human gut metagenome reads data set (MH0001) from the MetaHIT (Metagenomics of the Human Intestinal Tract) project [40] was used. It contains approximately 59 million reads, each 44 bp long; it was trimmed to remove low quality sequences.

Five soil metagenomics reads data sets with different size were taken from the GPGC project for benchmark purpose (see Table 1). These reads are from soil in Iowa region and they are filtered to make sure there are less than 30% Ns in the read and each read is longer than 30 bp. The exact data sets used for the paper are available on Amazon S3 and the instructions to acquire these data sets are available in the paper repository on github.com.

We also generated four short-read data sets to assess the false positive rate and miscount distribution. One is a subset of a real metagenomics data set from the MH0001 data set, above. The second consists of randomly generated reads. The third and fourth contain reads simulated from a random, 1 Mbp long genome. The third has a substitution error rate of 3%, and the fourth contains no errors. The four data sets were chosen to contain identical numbers of distinct 22-mers. The scripts necessary to regenerate these data are available in the paper repository on github.com.

### Count-Min Sketch implementation

We implemented the Count-Min Sketch data structure, a simple probabilistic data structure for counting distinct elements [12]. Our implementation uses 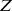 independent hash tables, each containing a prime number of counters 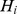. The hashing function for each hash table is fixed, and reversibly converts each DNA k-mer (for 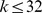) into a 64-bit number to which the modulus of the hash table size is applied. This provides 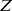 distinct hash functions.

To increment the count associated with a k-mer, the counter associated with the hashed k-mer in each of the 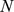 hash tables is incremented. To retrieve the count associated with a k-mer, the minimum count across all 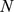 hash tables is chosen.

In this scheme, collisions are explicitly not handled, so the count associated with a k-mer may not be accurate. Because collisions only falsely *increment* counts, however, the retrieved count for any given k-mer is guaranteed to be no less than the correct count. Thus the counting error is one-sided.

### Hash function and khmer implementation

The current khmer hash function works only for 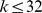 and converts DNA strings exactly into 64-bit numbers. However, any hash function would work. For example, a cyclic hash would enable khmer to count k-mers larger in size than 32; this would not change the scaling behavior of khmer at all, and is a planned extension.

By default khmer counts k-mers in DNA, i.e. strandedness is disregarded by having the hash function choose the lower numerical value for the exact hash of both a k-mer and its reverse complement. This behavior is configurable via a compile-time option.

### Comparing with other k-mer counting programs

We generated k-mer abundance distribution from five soil metagenomic reads data sets of different sizes using khmer, Tallymer, Jellyfish, DSK, BFCounter, KMC, Turtle and KAnalyze. If the software does not include function to generate k-mer abundance distribution directly, we output the frequency of each k-mer in an output file. We fixed k at 22 unless otherwise noted.

#### khmer

For khmer, we set hash table sizes to fix the false positive rate at either 1%, 5% or 20%, and used 8 threads in loading the data.

We did the khmer random-access k-mer counting benchmark with a custom-written Python script khmer-count-kmers which loaded the database file and then used the Python API to query each k-mer individually.

#### Tallymer

Tallymer is from the genometools package version 1.3.4. For the suffixerator subroutine we used: -dna -pl -tis -suf -lcp.

We use the mkindex subroutine to generate k-mer abundance distribution, we used: -mersize 22.

The Tallymer random access k-mer counting benchmark was done using the ‘tallymer search’ routine against both strands; see the script tallymer-search.sh.

#### Jellyfish

The Jellyfish version used was 1.1.10 and the multithreading option is set to 8 threads.

Jellyfish uses a hash table to store the k-mers and the size of the hash table can be modified by the user. When the specified hash table fills up, Jellyfish writes it to the hard disk and initializes a new hash table. Here we use a similar strategy as in [6] and chose the minimum size of the hash tables for Jellyfish so that all k-mers were stored in memory.

We ran Jellyfish with the options as below:

jellyfish count -m 22 -c 2 -C for k = 22.

The Jellyfish random access k-mer counting benchmark was performed using the ‘query’ routine and querying against both strands; see the script jelly-search.sh.

#### DSK

We ran DSK with default parameters with -histo option to generate k-mer abundance distribution. The DSK version used was 1.5031.

#### BFCounter

The BFcounter version used was 1.0 and the multithreading option is set to 8 threads.

We ran BFCounter count subroutine with the options as below:

BFCounter count -k 22 -t 8 -c 100000. -n option representing the estimated number of k-mers is adjusted to the different test data sets.

This subroutine produces the actual count of k-mers in input files.

We ran BFCounter dump subroutine with the options as below: BFCounter dump -k 22.

This subroutine can write k-mer occurrences into a tab-separated text file.

#### KMC

The KMC version used was 0.3. We ran both kmc and kmc_dump subroutines with default parameters.

#### Turtle

The Turtle version used was 0.3. We ran scTurtle32 with the multithreading option set to 8 threads and -n option representing expected number of frequent k-mers is adjusted to different test data sets.

#### KAnalyze

The KAnalyze version used was 0.9.3. We ran count subroutine with default parameters.

## References

[1] Marçais G, Kingsford C (2011) A fast, lock-free approach for efficient parallel counting of occurrences of k-mers. Bioinformatics 27: 764–770. 21217122 10.1093/bioinformatics/btr011 PMC3051319

[2] Kurtz S, Narechania A, Stein JC, Ware D (2008) A new method to compute K-mer frequencies and its application to annotate large repetitive plant genomes. BMC Genomics 9: 517. 18976482 10.1186/1471-2164-9-517 PMC2613927

[3] Metzker M (2010) Sequencing technologies - the next generation. Nat Rev Genet 11: 31–46. 19997069 10.1038/nrg2626

[4] Conway TC, Bromage AJ (2011) Succinct data structures for assembling large genomes. Bioinfor-matics 27: 479–86. 10.1093/bioinformatics/btq697 21245053

[5] Minoche AE, Dohm JC, Himmelbauer H (2011) Evaluation of genomic high-throughput sequencing data generated on illumina hiseq and genome analyzer systems. Genome Biol 12: R112. 22067484 10.1186/gb-2011-12-11-r112 PMC3334598

[6] Melsted P, Pritchard JK (2011) Efficient counting of k-mers in DNA sequences using a bloom filter. BMC bioinformatics 12: 333. 21831268 10.1186/1471-2105-12-333 PMC3166945

[7] Rizk G, Lavenier D, Chikhi R (2013) Dsk: k-mer counting with very low memory usage. Bioinfor- matics 29: 652–3. 10.1093/bioinformatics/btt020 23325618

[8] Deorowicz S, Debudaj-Grabysz A, Grabowski S (2013) Disk-based k-mer counting on a pc. BMC Bioinformatics 14: 160. 23679007 10.1186/1471-2105-14-160 PMC3680041

[9] Roy RS, Bhattacharya D, Schliep A (2014) Turtle: Identifying frequent k-mers with cache-efficient algorithms. Bioinformatics: Advance Access published March 10, 2014 : 10.1093/bioinformat-ics/btu132 24618471

[10] Audano P, Vannberg F (2014) Kanalyze: A fast versatile pipelined k-mer toolkit. Bioinformatics: Advance Access published March 18, 2014: 10.1093/bioinformatics/btu152 PMC4080738 24642064

[11] Howe AC, Jansson JK, Malfatti SA, Tringe SG, Tiedje JM, et al (2014) Tackling soil diversity with the assembly of large, complex metagenomes. Proc Natl Acad Sci U S A 111: 4904–9. 24632729 10.1073/pnas.1402564111 PMC3977251

[12] Cormode G, Muthukrishnan S (2005) An improved data stream summary: the count-min sketch and its applications. Journal of Algorithms 55: 58–75.

[13] Bloom BH (1970) Space/time trade-offs in hash coding with allowable errors. Commun ACM 13: 422–426.

[14] Malde K, O'Sullivan B (2009) Using bloom filters for large scale gene sequence analysis in haskell. In: Gill A, Swift T, editors, PADL. Springer, volume 5418 of *Lecture Notes in Computer Science*, pp. 183–194.

[15] Pell J, Hintze A, Canino-Koning R, Howe A, Tiedje JM, et al (2012) Scaling metagenome sequence assembly with probabilistic de bruijn graphs. Proc Natl Acad Sci U S A 109: 13272–7. 22847406 10.1073/pnas.1121464109 PMC3421212

[16] Jones DC, Ruzzo WL, Peng X, Katze MG (2012) Compression of next-generation sequencing reads aided by highly efficient de novo assembly. Nucleic Acids Res 40: e171. 22904078 10.1093/nar/gks754 PMC3526293

[17] Broder AZ, Mitzenmacher M (2003) Survey: Network applications of bloom filters: A survey. Internet Mathematics 1: 485–509.

[18] Fan L, Cao P, Almeida J, Broder AZ (2000) Summary cache: A scalable wide-area web cache sharing protocol. IEEE/ACM Trans Netw 8: 281–293.

[19] Estan C, Varghese G (2002) New directions in traffic measurement and accounting. In: SIGCOMM. ACM, pp. 323–336.

[20] Cohen S, Matias Y (2003) Spectral bloom filters. In: Halevy AY, Ives ZG, Doan A, editors, SIGMOD Conference. ACM, pp. 241–252.

[21] Brown CT, Howe A, Zhang Q, Pyrkosz AB, Brom TH (2012) A reference-free algorithm for com- putational normalization of shotgun sequencing data. arXiv: 1203.4802.

[22] Crusoe M, Edvenson G, Fish J, Howe A, McDonald E, et al (2014) The khmer software package: enabling efficient sequence analysis. URL 10.6084/m9.figshare.979190. PMC4608353 26535114

[23] McDonald E, Brown CT (2013) Working with big data in bioinformatics. In: Armstrong T, editor, The Performance of Open Source Applications, lulu.com, chapter 12. p. 151.

[24] Broder A, Mitzenmacher M (2004) Network applications of bloom filters: A survey. Internet mathematics 1: 485–509.

[25] Flajolet P, Fusy É, Gandouet O, Meunier F (2008) Hyperloglog: the analysis of a near-optimal cardinality estimation algorithm. DMTCS Proceedings.

[26] Chikhi R, Medvedev P (2014) Informed and automated k-mer size selection for genome assembly. Bioinformatics 30: 31–7. 23732276 10.1093/bioinformatics/btt310

[27] Medvedev P, Scott E, Kakaradov B, Pevzner P (2011) Error correction of high-throughput se- quencing datasets with non-uniform coverage. Bioinformatics 27: i137–41. 21685062 10.1093/bioinformatics/btr208 PMC3117386

[28] Pevzner PA, Tang H, Waterman MS (2001) An eulerian path approach to dna fragment assembly. Proc Natl Acad Sci U S A 98: 9748–53. 11504945 10.1073/pnas.171285098 PMC55524

[29] Li X, Waterman MS (2003) Estimating the repeat structure and length of dna sequences using l-tuples. Genome Res 13: 1916–22. 12902383 10.1101/gr.1251803 PMC403783

[30] Kelley DR, Schatz MC, Salzberg SL (2010) Quake: quality-aware detection and correction of sequencing errors. Genome Biol 11: R116. 21114842 10.1186/gb-2010-11-11-r116 PMC3156955

[31] Chitsaz H, Yee-Greenbaum J, Tesler G, Lombardo M, Dupont C, et al (2011) Efficient de novo assembly of single-cell bacterial genomes from short-read data sets. Nat Biotechnol 29: 915–21. 21926975 10.1038/nbt.1966 PMC3558281

[32] Haas BJ, Papanicolaou A, Yassour M, Grabherr M, Blood PD, et al (2013) De novo transcript sequence reconstruction from rna-seq using the trinity platform for reference generation and analysis. Nat Protoc 8: 1494–512. 23845962 10.1038/nprot.2013.084 PMC3875132

[33] Brown CT (2012) What does trinity's in silico normalization do? URL 10.6084/m9.figshare.98198.

[34] Zerbino DR, Birney E (2008) Velvet: algorithms for de novo short read assembly using de bruijn graphs. Genome Res 18: 821–9. 18349386 10.1101/gr.074492.107 PMC2336801

[35] Rusu F, Dobra A (2008) Sketches for size of join estimation. ACM Transactions on Database Systems 33: 1–46.

[36] Cormode G, Muthukrishnan S (2005) Summarizing and mining skewed data streams. In: Kargupta H, Srivastava J, Kamath C, Goodman A, editors, SDM. SIAM, pp. 44–55.

[37] Luo W, Friedman MS, Shedden K, Hankenson KD, Woolf PJ (2009) Gage: generally applicable gene set enrichment for pathway analysis. BMC Bioinformatics 10: 161. 19473525 10.1186/1471-2105-10-161 PMC2696452

[38] Howe AC, Pell J, Canino-Koning R, Mackelprang R, Tringe S, et al. (2012) Illumina sequencing artifacts revealed by connectivity analysis of metagenomic datasets. arXiv: 1212.0159.

[39] Pérez F, Granger B (2007) Ipython: A system for interactive scientific computing. Computing in Science Engineering 9: 21–29.

[40] Qin J, Li R, Raes J, Arumugam M, Burgdorf KS, et al (2010) A human gut microbial gene catalogue established by metagenomic sequencing. Nature 464: 59–65. 20203603 10.1038/nature08821 PMC3779803

